# Poor tumor differentiation is an independent adverse prognostic variable in patients with locally advanced oral cavity cancer––Comparison with pathological risk factors according to the NCCN guidelines

**DOI:** 10.1002/cam4.4195

**Published:** 2021-09-17

**Authors:** Li‐Yu Lee, Chien‐Yu Lin, Nai‐Ming Cheng, Chi‐Ying Tsai, Chuen Hsueh, Kang‐Hsing Fan, Hung‐Ming Wang, Chia‐Hsun Hsieh, Shu‐Hang Ng, Chih‐Hua Yeh, Chih‐Hung Lin, Chung‐Kan Tsao, Tuan‐Jen Fang, Shiang‐Fu Huang, Li‐Ang Lee, Chung‐Jan Kang, Ku‐Hao Fang, Yu‐Chien Wang, Wan‐Ni Lin, Li‐Jen Hsin, Tzu‐Chen Yen, Chun‐Ta Liao

**Affiliations:** ^1^ Department of Pathology Chang Gung Memorial Hospital and Chang Gung University Taoyuan Taiwan, Republic of China; ^2^ Department of Radiation Oncology Chang Gung Memorial Hospital and Chang Gung University Taoyuan Taiwan, Republic of China; ^3^ Department of Nuclear Medicine and Molecular Imaging Center Chang Gung Memorial Hospital and Chang Gung University Taoyuan Taiwan, Republic of China; ^4^ Department of Oral and Maxillofacial Surgery Chang Gung Memorial Hospital Chang Gung University Taoyuan Taiwan, Republic of China; ^5^ Department of Medical Oncology Chang Gung Memorial Hospital and Chang Gung University Taoyuan Taiwan, Republic of China; ^6^ Department of Diagnostic Radiology Chang Gung Memorial Hospital and Chang Gung University Taoyuan Taiwan, Republic of China; ^7^ Department of Plastic and Reconstructive Surgery Chang Gung Memorial Hospital and Chang Gung University Taoyuan Taiwan, Republic of China; ^8^ Department of Otorhinolaryngology, Head and Neck Surgery Chang Gung Memorial Hospital and Chang Gung University Taoyuan Taiwan, Republic of China; ^9^ Particle Physics and Beam Delivery Core Laboratory Institute for Radiological Research Chang Gung Memorial Hospital and Chang Gung University Taoyuan Taiwan, Republic of China

**Keywords:** histopathological risk factors, oral cavity, prognosis, squamous cell carcinoma, tumor differentiation

## Abstract

**Methods:**

We sought to compare the prognostic impact of tumor differentiation with respect to adverse risk factors (RFs) identified by the National Comprehensive Cancer Network (NCCN) guidelines––including extranodal extension (ENE), positive/close margins, perineural invasion, lymphatic invasion, and vascular invasion––in patients with locally advanced oral cavity squamous cell carcinoma (OCSCC).

**Results:**

Between 1996 and 2018, 1179 consecutive patients with first primary pT3–4 OCSCC were included. A three‐level grading system was adopted––in which the final classification was assigned according to the most prevalent tumor grade. We identified 382/669/128 patients with well/moderately/poorly differentiated tumors, respectively. Compared with well/moderately differentiated tumors, poorly differentiated OCSCC had a higher prevalence of the following variables: female sex (4%/6%/11%), ENE, (14%/36%/61%), positive margins (0.5%/2%/4%), close margins (10%/14%/22%), perineural invasion (22%/50%/63%), lymphatic invasion (2%/9%/17%), vascular invasion (1%/4%/10%), and adjuvant therapy (64%/80%/87%). The 5‐year rates of patients with well/moderately/poorly differentiated OCSCC were as follows: local control (LC, 85%/82%/84%, *p *= 0.439), neck control (NC, 91%/83%/70%, *p *< 0.001), distant metastases (DM, 6%/18%/40%, *p *< 0.001), disease‐free survival (DFS, 78%/63%/46%, *p *< 0.001), disease‐specific survival (DSS, 85%/71%/49%, *p *< 0.001), and overall survival (OS, 68%/55%/39%, *p *< 0.001). Multivariable analysis identified the following variables as independent prognosticators for 5‐year outcomes: ENE (LC/NC/DM/DFS/DSS/OS), poorly differentiated tumors (NC/DM/DFS/DSS/OS), positive margins (LC/DFS), lymphatic invasion (DFS/DSS/OS), perineural invasion (DM), and age ≥65 years (OS).

**Conclusions:**

In addition to ENE, poor tumor differentiation was identified as the second most relevant adverse RF for patients with pT3–4 OCSCC. We suggest that the NCCN guidelines should include poor tumor differentiation as an adverse RF to refine and tailor clinical management.

## Graphical abstract

Final tumor grading of oral cavity squamous cell carcinoma (OCSCC) is assigned according to the most prevalent grade. Relapses of poorly differentiated OCSCC tended to occur regionally and distantly. Poor tumor differentiation was the second most relevant adverse risk factor after extranodal extension. The NCCN guidelines should include poor differentiation as an adverse risk factor.

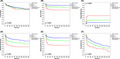

## 1 INTRODUCTION

Surgery––either with or without adjuvant therapy––remains the mainstay of treatment for oral cavity squamous cell carcinoma (OCSCC).^1^ Clinical outcomes of patients with OCSCC are chiefly driven by locoregional control, and radical surgical excision is of paramount importance for achieving a favorable prognosis.^2^ As for neck control, level I–III and I–V neck dissections (NDs) are recommended for patients with cN0 and cN+ diseases, respectively. Besides clinical and imaging parameters, a number of histopathology variables––which reflect the tumor's biological behavior––are deemed of prognostic importance in OCSCC. For example, extranodal extension (ENE) is known to portend an increased risk of local, regional, and distant relapses.

The National Comprehensive Cancer Network (NCCN) treatment guidelines have identified several adverse histopathological parameters for patients with OCSCC––including ENE, positive margins, margins <5 mm (close margins), pN2–3 disease, pN1 at level IV/V, pT3–4 tumors, perineural invasion, lymphatic invasion, and vascular invasion.^3^ The presence of these variables poses an indication for postoperative adjuvant therapy.^3^ Notably, the NCCN guidelines does not include poor tumor differentiation as an independent unfavorable prognostic factor for OCSCC. However, our clinical experience suggests that patients with poorly differentiated OCSCC are at an increased risk for nodal metastases compared with those with well or moderately differentiated tumors. In this scenario, poorly differentiated OCSCC is expected to have poor outcomes in terms of neck control (NC), distant metastases (DM), and survival rates. Nonetheless, the prognostic impact of tumor differentiation in patients OCSCC is still a matter of ongoing debate, and the published literature in the field is conflicting.^4^, ^5^, ^6^, ^7^, ^8^, ^9^, ^10^, ^11^, ^12^, ^13^, ^14^, ^15^, ^16^, ^17^, ^18^, ^19^, ^20^, ^21^, ^22^ Some authors have also suggested to replace tumor differentiation with novel prognostic scoring systems.^9^, ^10^, ^11^, ^12^, ^13^, ^14^, ^17^, ^20^, ^22^ To the best of our knowledge, no large cohort study has specifically analyzed the prognostic impact of tumor differentiation in relation to different clinical outcomes––including local, regional, and distant control––in patients with OCSCC.

Therefore, we designed the current retrospective study to compare the prognostic significance of tumor differentiation with respect to adverse risk factors (RFs) identified by the NCCN guidelines. To this aim, we specifically focused on patients with locally advanced tumors (pT3–4 disease).

## 2 PATIENTS AND METHODS

### 2.1 Study setting

The study protocol followed the tenets set forth by the Helsinki declaration and was approved by the local institutional review board (CGMH 101‐4457B, 202100048B0). We retrospectively reviewed the clinical records of all consecutive patients with first primary pT3–4 OCSCC (*n* = 1179) who were consecutively referred to the Chang Gung Memorial Hospital between January 1996 and December 2018. Owing to the retrospective study design, the need for informed consent was waived. All patients who were scheduled to undergo radical surgery––either with (*n* = 1161) or without (*n* = 18) NDs––received a thorough presurgical evaluation and staging workup as previously described.^23^, ^24^, ^25^ Clinicopathological RFs were prospectively collected by investigators who were blinded to clinical endpoints. All histopathological variables were independently reviewed by two experienced head and neck pathologists using a dedicated checklist. Because of the prospective data collection for both tumor depth of invasion (DOI) and ENE,^26^ patients were staged according to the AJCC staging manual, eighth edition.^27^

### 2.2 Surgery and adjuvant therapy

Primary tumors were removed with ≥1 cm margins (both peripheral and deep margins). Patients with cN+ disease underwent level I–IV or I–V NDs, whereas cN‐ patients received level I–III NDs. Patients who harbored pathological RFs were generally treated with postoperative radiotherapy (RT, 60 Gy) or concurrent chemoradiotherapy (CCRT, 66 Gy).^28^, ^29^, ^30^ RFs were assessed using the NCCN guidelines until 2008^3^; thereafter, the Chang Gung Memorial Hospital (CGMH) guidelines were adopted.^1^ The radiation field consisted of the entire tumor bed area (with 1‐ to 2‐cm margins) and regional lymphatics. We used the following chemotherapy regimens: intravenous cisplatin 50 mg/m^2^ biweekly plus daily oral tegafur 800 mg and leucovorin 60 mg, cisplatin 40 mg/m^2^ weekly, or cisplatin 100 mg/m^2^ every 3 weeks.^30^ Patients who refused the proposed approaches or had unexpected evidence of disease stage modifications in the postoperative period were treated with surgery alone.

### 2.3 Follow‐up schedule and data collection

Postoperative follow‐up was performed on a regular basis at different intervals in relation to the severity of the disease, as follows: every 1–3 months during the first postoperative year; every 2–4 months during the second year; and every 4–6 months between the third and the fifth years. Patients who survived more than 5 years after surgery were followed every 6–12 months. Data pertaining to clinical events––including local control (LC), NC, DM, disease‐free survival (DFS), disease‐specific survival (DSS), and overall survival (OS)––were updated at each follow‐up visit.

### 2.4 Primary tumor histology

**FIGURE 1:**
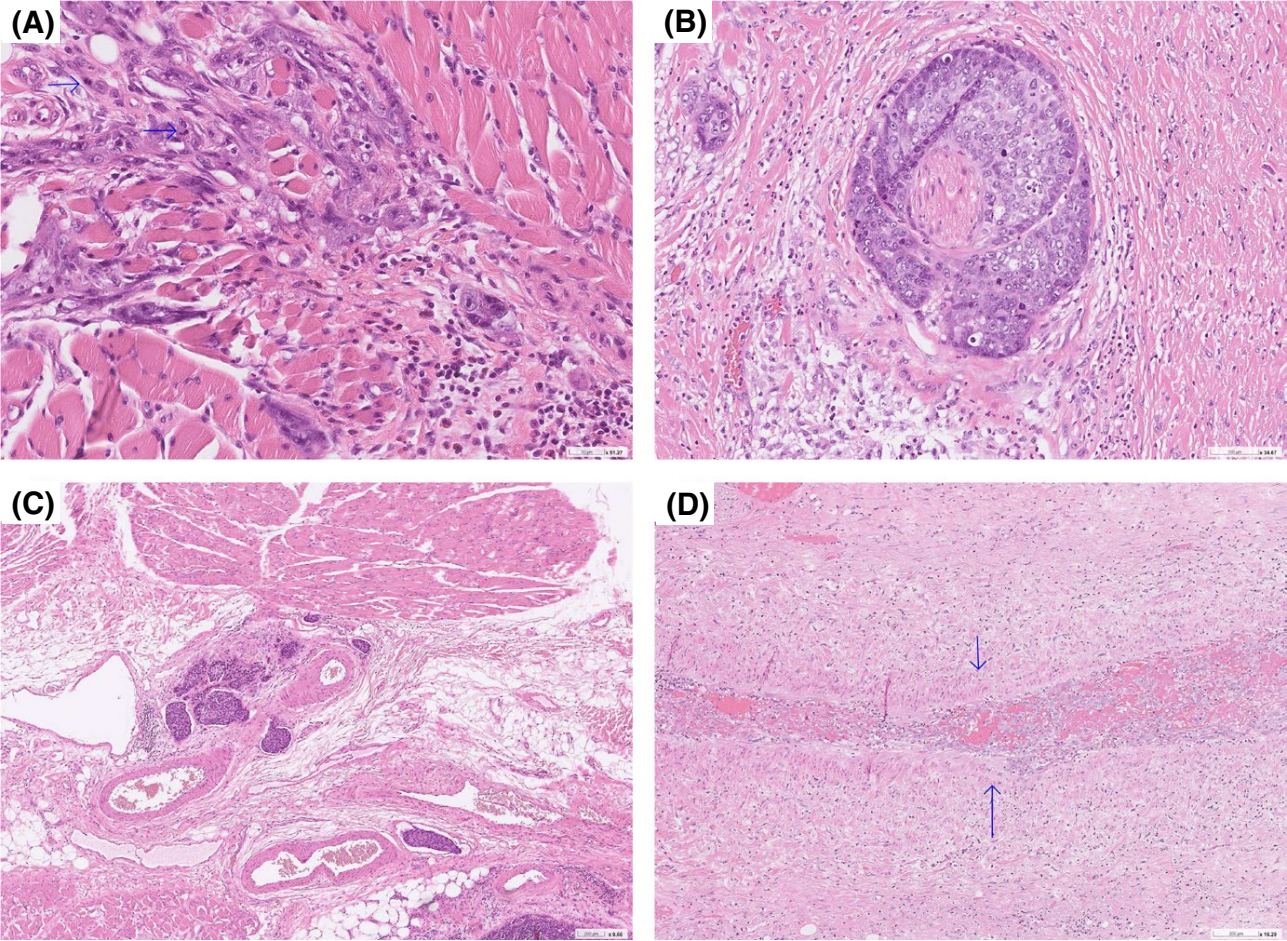
Representative histological findings of poorly differentiated oral cavity squamous cell carcinoma. Panel A: tumor cells––characterized by a high nucleus‐to‐cytoplasm ratio, marked nuclear pleomorphism, and high mitotic activity (the arrows indicate mitoses)––infiltrating the skeletal muscle tissue; Panel B: perineural invasion. Panel C: lymphatic invasion. Panel D: vascular invasion (the arrows indicate the smooth muscle wall)

Primary tumor sections were obtained from at least four paraffin blocks. A three‐level grading system was adopted––in which the final classification was assigned according to the most prevalent tumor grade according to the CAP Cancer Reporting Protocols recommendations.^31^ Figure 1 illustrates representative histological findings of poorly differentiated OCSCC (Figure 1A–D). The final pathological report also included the following variables: tumor thickness, DOI, margin status, perineural invasion, lymphatic invasion, and vascular invasion.

### 2.5 Statistical analysis

All patients received follow‐up examinations for at least 24 months after surgery or until death. Follow‐up visits were continued until December 2020. Descriptive statistics are given as frequencies, percentages, means, medians, ranges, and standard deviations (SD). The study endpoints included the 5‐year rates of LC, NC, DM, DFS, DSS, and OS. The time elapsed from the date of surgery to the date of event was calculated for each endpoint of interest. Time‐dependent outcomes were analyzed by the Kaplan–Meier method and compared with the log‐rank test. Univariate analysis (UVA) and multivariable Cox regression analysis (MVA) were applied to assess the associations between RFs and clinical outcomes. Any variable that was included in UVA was entered as a covariate into the multivariable model. Results of UVA and MVA are presented as hazard ratios (HRs) with their 95% confidence intervals (CIs). All analyses were two‐tailed, and *p* values <0.05 were considered as statistically significant.

## 3 RESULTS

### 3.1 General characteristics of patients with pT3–4 OCSCC according to tumor differentiation

Most of the study patients were men (94.4%) and aged <65 years (86.7%). Of the 1179 pT3–4 OCSCC patients, there were 382 (32.4%), 669 (56.7%), and 128 (10.9%) patients with well, moderately, and poorly differentiated tumors, respectively. Table 1 depicts the general characteristics of the study participants stratified according to the three tumor differentiation categories. Compared with patients harboring well differentiated and moderately differentiated tumors, those with poorly differentiated OCSCC showed a significantly higher prevalence of the following variables: female sex (3.7% vs. 5.7% vs. 10.9%, *p *= 0.008), pN2–3 disease (20.3% vs. 44.1% vs. 68.5%, *p* < 0.001), ENE (14.2% vs. 36.4% vs. 61.4%, *p* < 0.001), positive/close margins (<5 mm) (0.5%/9.9% vs. 2.1%/14.3% vs. 4.0%/21.6%, *p *< 0.001), perineural invasion (22.3% vs. 50.1% vs. 63.3%, *p* < 0.001), lymphatic invasion (2.6% vs. 9.3% vs. 17.2%, *p *< 0.001), vascular invasion (1.3% vs. 4.0% vs. 10.2%, *p* < 0.001), and treatment with adjuvant therapy (64.1% vs. 79.8% vs. 86.7%, *p *< 0.001).

**TABLE 1:** General characteristics of patients with pT3–4 oral cavity squamous cell carcinoma (*n* = 1179) stratified according to the presence of well, moderately, and poorly differentiated tumors

Characteristic (*n*, %)	Well differentiated OCSCC (*n* = 382)	Moderately differentiated OCSCC (*n* = 669)	Poorly differentiated OCSCC (*n* = 128)	*p*
	*n* (%)	*n* (%)	*n* (%)	
Sex				0.008
Men (1113, 94.4)	368 (96.3)	631 (94.3)	114 (89.1)	
Women (66, 5.6)	14 (3.7)	38 (5.7)	14 (10.9)	
Age (years)				0.710
<65 (1022, 86.7)	333 (87.2)	581 (86.8)	108 (84.4)	
≥65 (157, 13.3)	49 (12.8)	88 (13.2)	20 (15.6)	
Pathologic N status				<0.001
pN0–1 (706, 60.8%)	294 (79.7)	372 (55.9)	40 (31.5)	
pN2–3 (455, 39.2%)	75 (20.3)	293 (44.1)	87 (68.5)	
Extranodal extension^a^				<0.001
No (786, 67.9)	314 (85.8)	423 (63.6)	49 (38.6)	
Yes (372, 32.1)	52 (14.2)	242 (36.4)	78 (61.4)	
Margin status^a^				0.001
≥5 mm (986, 84.6)	335 (89.6)	556 (83.6)	95 (74.4)	
<5 mm (159, 13.6)	37 (9.9)	95 (14.3)	27 (21.6)	
Positive (21, 1.8)	2 (0.5)	14 (2.1)	5 (4.0)	
Perineural invasion				<0.001
No (678, 57.5)	297 (77.7)	334 (49.9)	47 (36.7)	
Yes (501, 42.5)	85 (22.3)	335 (50.1)	81 (63.3)	
Lymphatic invasion^a^				<0.001
No (1084, 92.0)	371 (97.4)	607 (90.7)	106 (82.8)	
Yes (94, 8.0)	10 (2.6)	62 (9.3)	22 (17.2)	
Vascular invasion^a^				<0.001
No (1133, 96.2)	376 (98.7)	642 (96.0)	115 (89.8)	
Yes (45, 3.8)	5 (1.3)	27 (4.0)	13 (10.2)	
Treatment modality				<0.001
S alone (289, 24.5)	137 (35.9)	135 (20.2)	17 (13.3)	
S plus RT/CCRT (890, 75.5)	245 (64.1)	534 (79.8)	111 (86.7)	

Abbreviations: CCRT, concurrent chemoradiotherapy; OCSCC, oral cavity squamous cell carcinoma; RT, radiotherapy;S, surgery.

a Unavailable data: extranodal extension (*n* = 21, included three unknown and 18 who did not undergo neck dissection), margin status (*n* = 13), lymphatic invasion (*n* = 1), and vascular invasion (*n* = 1).

### 3.2 Five‐year survival rates of patients with pT3–4 OCSCC in the entire cohort study

The median follow‐up time of the entire cohort of patients with pT3–4 OCSCC was 58 months (mean: 77 months; SD: 69 months; range: 1–286 months). The median follow‐up time for patients who survived was 111 months (mean: 121 months; SD, 65 months; range, 24–286 months). The 5‐year rates observed in our cohort were as follows: LC, 83%; NC, 84%; DM, 16%; DFS, 66%; DSS, 74%; and OS, 58%, respectively.

### 3.3 Five‐year survival rates of patients with pT3–4 OCSCC according to tumor differentiation

**FIGURE 2:**
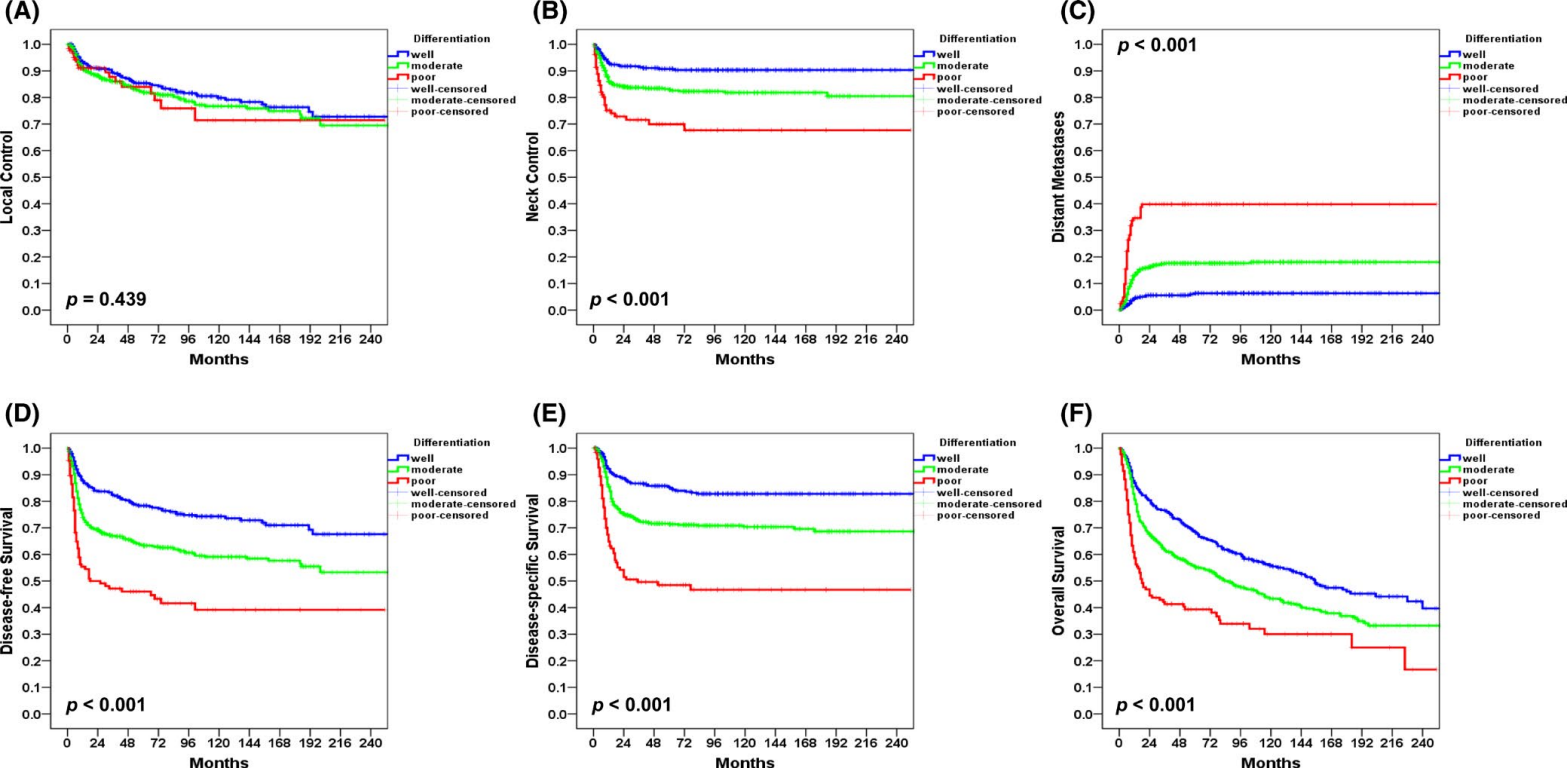
Kaplan–Meier plots of 5‐year local control (panel A), neck control (panel B), distant metastases (panel C), disease‐free survival (panel D), disease‐specific survival (panel E), and overall survival (panel F) in patients with poorly, moderately, and well differentiated oral cavity squamous cell carcinoma

The 5‐year rates of patients with well differentiated, moderately differentiated, and poorly differentiated OCSCC were as follows: LC, 85%/82%/84%, *p* = 0.439; NC, 91%/83%/70%, *p *< 0.001; DM, 6%/18%/40%, *p* < 0.001; DFS, 78%/63%/46%, *p *< 0.001; DSS, 85%/71%/49%, *p* < 0.001; and OS, 68%/55%/39%, *p* < 0.001; respectively (Figure 2A–F). Thus, the 5‐year outcomes of poorly differentiated OCSCC were less favorable than those observed in patients with moderately differentiated and well differentiated tumors––the only exception being local control.

### 3.4 Five‐year outcomes according to tumor differentiation versus adverse pathological risk factors according to the NCCN guidelines

The following variables were analyzed in relation to different 5‐year outcomes: differentiation (poorly differentiated vs. well/moderately differentiated tumors), sex (men vs. women), age at onset (≥65 vs. <65 years), ENE (present vs. absent), margin status (positive margins vs. <5 vs. ≥5 mm), perineural invasion (present vs. absent), lymphatic invasion (present vs. absent), vascular invasion (present vs. absent), and treatment modality (surgery plus adjuvant therapy vs. surgery).

Kaplan–Meier curves identified the following variables as significant RFs for 5‐year outcomes (Table 2): pN2–3 disease (LC/NC/DM/DFS/DSS/OS), ENE (LC/NC/DM/DFS/DSS/OS), perineural invasion (LC/NC/DM/DFS/DSS/OS), lymphatic invasion (LC/NC/DM/DFS/DSS/OS), poorly differentiated OCSCC (NC/DM/DFS/DSS/OS), vascular invasion (NC/DM/DFS/DSS/OS), margins status (LC/DM/DFS/DSS/OS), and age ≥65 years (OS).

**TABLE 2:** Five‐year local control, neck control, distant metastasis, and survival rates in patients with pT3–4 oral cavity squamous cell carcinoma (*n* = 1179)

Characteristics (*n*, %)	5‐year LC % (*n* event)	*p*	5‐year NC % (*n* event)	*p*	5‐year DM % (*n* event)	*p*	5‐year DFS % (*n* event)	*p*	5‐year DSS % (*n* event)	*p*	5‐year OS % (*n* event)	*p*
Sex		0.989		0.270		0.786		0.428		0.632		0.639
Men (1113, 94.4)	83 (151)		84 (159)		16 (168)		66 (354)		73 (278)		58 (461)	
Women (66, 5.6)	83 (9)		82 (11)		15 (9)		71 (17)		76 (14)		58 (27)	
Age of disease onset, years		0.033		0.730		0.279		0.087		0.165		0.022
<65 (1022, 86.7)	82 (147)		84 (149)		17 (159)		66 (332)		73 (263)		59 (415)	
≥65 (157, 13.3)	89 (13)		85 (21)		14 (18)		72 (39)		79 (29)		53 (73)	
Pathological N status		0.012		<0.001		<0.001		<0.001		<0.001		<0.001
pN0–1 (706, 60.8)	86 (88)		92 (53)		6 (42)		78 (144)		87 (90)		72 (195)	
pN2–3 (455, 39.2)	78 (70)		71 (114)		34 (134)		47 (223)		51 (199)		36 (287)	
Extranodal extension		0.005		<0.001		<0.001		<0.001		<0.001		<0.001
No (786, 67.9)	85 (100)		90 (75)		7 (56)		76 (177)		84 (118)		69 (239)	
Yes (372, 32.1)	77 (58)		71 (92)		37 (120)		44 (190)		48 (171)		34 (242)	
Differentiation		0.498		<0.001		<0.001		<0.001		<0.001		<0.001
Well‐moderate (1051, 89.1)	83 (146)		86 (137)		13 (131)		69 (307)		76 (232)		60 (411)	
Poor (128, 10.9)	84 (14)		70 (33)		40 (46)		46 (64)		49 (60)		39 (77)	
Margin status		0.001		0.894		0.011		0.001		0.002		0.005
≥5 mm (986, 84.6)	86 (116)		85 (136)		15 (137)		69 (287)		75 (225)		60 (389)	
<5 mm (159, 13.6)	79 (25)		85 (20)		21 (32)		60 (60)		71 (44)		52 (73)	
Positive (21, 1.8)	66 (6)		86 (3)		29 (6)		35 (11)		52 (10)		28 (13)	
Perineural invasion		0.010		<0.001		<0.001		<0.001		<0.001		<0.001
No (678, 57.5)	86 (76)		88 (74)		11 (68)		73 (167)		80 (125)		63(247)	
Yes (501, 42.5)	79 (84)		79 (96)		24 (109)		57 (204)		64 (167)		51 (241)	
Lymphatic invasion		0.032		<0.001		<0.001		<0.001		<0.001		<0.001
No (1084, 92.0)	84 (142)		86 (144)		14 (145)		69 (319)		76 (247)		60 (420)	
Yes (94, 8.0)	66 (17)		67 (25)		39 (32)		36 (51)		43 (44)		28 (67)	
Vascular invasion		0.086		0.003		<0.001		<0.001		<0.001		<0.001
No (1133, 96.2)	84 (150)		85 (157)		15 (163)		68 (346)		74 (271)		59 (458)	
Yes (45, 3.8)	69 (9)		68 (12)		36 (14)		39 (24)		50 (20)		35 (29)	
Treatment modality		0.239		0.130		<0.001		<0.001		<0.001		<0.001
S alone (289, 24.5)	88 (30)		87 (35)		5 (14)		81 (52)		87 (36)		73 (76)	
S plus RT/CCRT (890, 75.5)	81 (130)		83 (135)		20 (163)		62 (319)		69 (256)		53 (412)	

Abbreviations: CCRT, concurrent chemoradiotherapy; DFS, disease‐free survival; DM, distant metastases; DSS, disease‐specific survival; LC, local control; NC, neck control; OS, overall survival; RT, radiotherapy; S, surgery.

### 3.5 Multivariable cox regression analysis according to the presence or absence of ENE alone or the ENE/pN classification

In light of the overlap between the presence of ENE and the pN classification, we conducted two separated analyses focusing on the prognostic significance of ENE alone versus both ENE and the pN classification. We initially assigned the following reference categories (HR = 1): female sex, age <65 years, well and moderately differentiated tumors, pN0–1 disease, absence of ENE, margins ≥5 mm, absence of perineural invasion, absence of lymphatic invasion, absence of vascular invasion, and treatment with surgery alone. On multivariable analyses (presence or absence of ENE alone) with a forward stepwise selection procedure, we identified the following RFs as independently associated with 5‐year outcomes: ENE (LC/NC/DM/DFS/DSS/OS), poorly differentiated tumors (NC/DM/DFS/DSS/OS), positive margins (LC/DFS), lymphatic invasion (DFS/DSS/OS), perineural invasion (DM), and age ≥65 years (OS) (Table 3). On multivariable analyses (ENE/pN classification) with a forward stepwise selection procedure, we identified the following RFs as independently associated with 5‐year outcomes: ENE (LC/DM/DFS/OS), pN2–3 disease (NC/DM/DFS/DSS), poorly differentiated tumors (NC/DM/DFS/DSS/OS), positive margins (LC/DFS/DSS), lymphatic invasion (DFS/OS), perineural invasion (DM/DSS), and age ≥65 years (OS) (Table 4).

**TABLE 3:** Multivariable analyses of risk factors for 5‐year local control, neck control, distant metastasis, and survival rates in patients with pT3–4 oral cavity squamous cell carcinoma according to the extranodal extension status (*n* = 1179)

Risk factor	Local control		Neck control		Distant metastases		Disease‐free survival		Disease‐specific survival		Overall survival	
	HR (95% CI)	*p*	HR (95% CI)	*p*	HR (95% CI)	*p*	HR (95% CI)	*p*	HR (95% CI)	*p*	HR (95% CI)	*p*
Extranodal extension (vs. absence)	1.459 (1.067–1.995)	0.018	2.980 (2.168–4.096)	<0.001	4.617 (3.304–6.454)	<0.001	2.453 (1.991–3.053)	<0.001	2.998 (2.304–3.901)	<0.001	2.062 (1.720–2.473)	<0.001
Poor differentiation (vs. well/moderate)		ns	1.904 (1.285–2.822)	0.001	2.241 (1.581–3.176)	<0.001	1.689 (1.287–2.218)	<0.001	1.824 (1.360–2.447)	<0.001	1.359 (1.069–1.727)	0.012
Positive margins (vs. ≥5 mm)	3.622 (1.591–8.246)	0.002		ns		ns	2.350 (1.285–4.299)	0.006		ns		ns
Margins <5 mm (vs. ≥5 mm)		ns		ns		ns		ns		ns		ns
Lymphatic invasion (vs. absence)		ns		ns		ns	1.554 (1.136–2.126)	0.006	1.428 (1.019–2.001)	0.039	1.479 (1.139–1.922)	0.003
Perineural invasion (vs. absence)		ns		ns	1.440 (1.049–1.976)	0.024		ns		ns		ns
Age ≥65 years (vs. <65 years)		ns		ns		ns		ns		ns	1.348 (1.073–1.693)	0.010
S plus RT/CCRT (vs. S alone)		ns		ns		ns		ns	1.501 (1.005–2.242)	0.047	1.427 (1.138–1.790)	0.002

Abbreviations: CCRT, chemoradiotherapy; CI, confidence interval; HR, hazard ratio; ns, not significant; RT, radiotherapy; S, surgery.

**TABLE 4:** Multivariable analyses of risk factors for 5‐year local control, neck control, distant metastasis, and survival rates in patients with pT3–4 oral cavity squamous cell carcinoma according to the extranodal extension status and pN classification (*n* = 1179)

Risk factor	Local control		Neck control		Distant metastases		Disease‐free survival		Disease‐specific survival		Overall survival	
	HR (95% CI)	*p*	HR (95% CI)	*p*	HR (95% CI)	*p*	HR (95% CI)	*p*	HR (95% CI)	*p*	HR (95% CI)	*p*
Extranodal extension (vs. absence)	1.455 (1.064–1.990)	0.019		ns	1.810 (1.036–3.162)	0.037	1.498 (1.017–2.207)	0.041		ns	2.062 (1.720–2.473)	<0.001
pN2–3 (vs. pN0–1)		ns	3.544 (2.547–4.929)	<0.001	3.098 (1.691–5.676)	<0.001	1.774 (1.198–2.627)	0.004	3.696 (2.858–4.779)	<0.001		ns
Poor differentiation (vs. well/moderate)		ns	1.849 (1.251–2.733)	0.002	2.198 (1.552–3.112)	<0.001	1.663 (1.267–2.183)	<0.001	1.881 (1.404–2.519)	<0.001	1.359 (1.069–1.727)	0.012
Positive margins (vs. ≥5 mm)	3.622 (1.591–8.246)	0.002		ns		ns	2.407 (1.315–4.406)	0.004	2.331 (1.235–4.400)	0.009		ns
Margin <5 mm (vs. ≥5 mm)		ns		ns		ns		ns		ns		ns
Lymphatic invasion (vs. absence)		ns		ns		ns	1.472 (1.076–2.015)	0.016		ns	1.479 (1.139–1.922)	0.003
Perineural invasion (vs. absence)		ns		ns	1.401 (1.022–1.921)	0.036		ns	1.333 (1.047–1.697)	0.020		ns
Age ≥65 years (vs. <65 years)		ns		ns		ns		ns		ns	1.348 (1.073–1.693)	0.010
S plus RT/CCRT (vs. S alone)		ns		ns		ns		ns		ns	1.427 (1.138–1.790)	0.002

Abbreviations: CCRT, chemoradiotherapy; CI, confidence interval; HR, hazard ratio; ns, not significant; RT, radiotherapy; S, surgery.

When both ENE and the pN classification (pN2–3 vs. pN0–1) were included in the analysis, the prognostic significance of poor differentiation did not appreciably change. ENE and the presence of pN2–3 disease were identified as independent adverse RFs for different outcomes.

### 3.6 Five‐year outcomes according to the presence of ENE and poorly differentiated OCSCC

**FIGURE 3:**
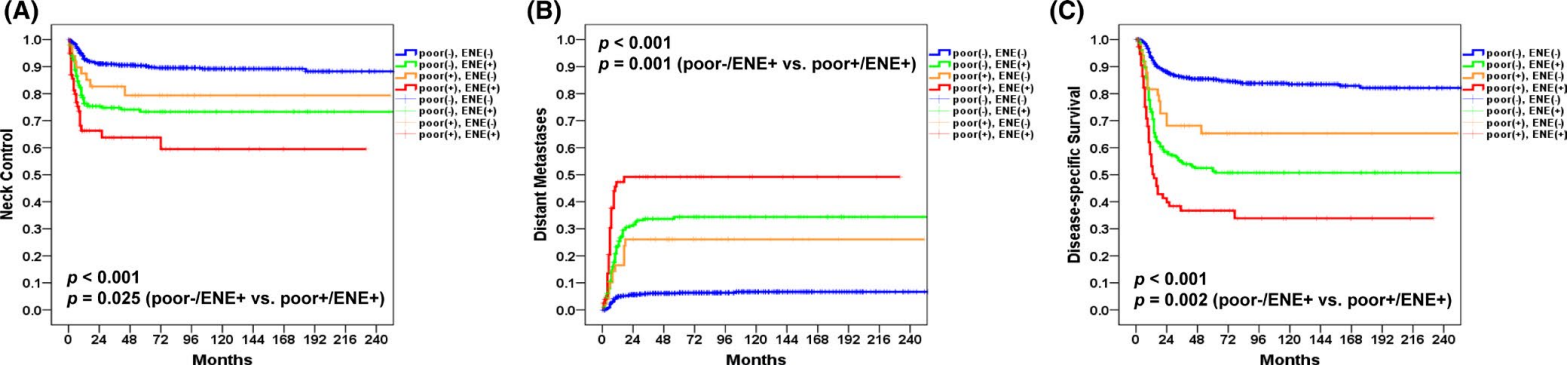
Kaplan–Meier plots of 5‐year neck control (panel A), distant metastases (panel B), and disease‐specific survival (panel C) in (1) patients with ENE and poorly differentiated tumors, (2) patients with ENE and well/moderately differentiated tumors, (3) patients with poorly differentiated tumors and no ENE, and (4) patients with well/moderately differentiated tumors and no ENE

According to the AJCC Staging Manual, eight edition, pN3b disease is defined by the presence of both pN2 disease (according to AJCC Staging Manual, seventh edition) and ENE. In this study, no cases of pN3a disease (defined by the presence of a single lymph node metastasis >6 cm in the absence of ENE) were identified. Therefore, all cases classified as pN3 had pN3b disease. Notably, 82% (372/455) of patients with pN2–3 disease had evidence of ENE. In this scenario, ENE appeared as the main driver of lymph node metastases. In addition to ENE, poor tumor differentiation was identified as the second most relevant adverse RF (Table 3). Therefore, we analyzed the 5‐year outcomes in the following four specific subgroups: (1) patients with ENE and poorly differentiated tumors, (2) patients with ENE and well/moderately differentiated tumors, (3) patients with poorly differentiated tumors and no ENE, and 4) patients with well/moderately differentiated tumors and no ENE. The 5‐year rates in the four subgroups were as follows: NC, 64%/73%/79%/90%, *p *< 0.001 (Figure 3A); DM, 49%/34%/26%/6%, *p *< 0.001 (Figure 3B); DFS, 37%/46%/60%/77%, *p *< 0.001; and DSS, 37%/51%/65%/85%, respectively, *p* < 0.001 (Figure 3C). Specifically, the 5‐year outcomes of patients with ENE and poorly differentiated tumors versus ENE and well/moderately differentiated tumors were as follows: NC, 64% versus 73%, *p *= 0.025 (Figure 3A); DM, 49% versus 34%, *p* = 0.001 (Figure 3B); DFS, 37% versus 46%, *p* = 0.009; and DSS, 37% versu*s* 51%, respectively, *p* = 0.002 (Figure 3C). Thus, the concomitant presence of poorly differentiated tumors and ENE was associated with less favorable outcomes compared with the presence of ENE alone.

## 4 DISCUSSION

The results of our study demonstrate that patients with pT3–4 OCSCC and poorly differentiated tumors tended to relapse at regional and distal––rather than local––sites. Notably, the presence of poor tumor differentiation had an adverse impact on all survival endpoints (i.e., DFS, DSS, and OS). In univariate Kaplan–Meier analyses, we identified three variables (i.e., ENE, perineural invasion, and lymphatic invasion) that had an unfavorable prognostic significance for all endpoints (i.e., LC/NC/DM/DFS/DSS/OS; Table 2). However, only ENE was retained as an independent adverse prognosticator for all outcomes after adjustment for potential confounders in MVA. It should be noted, however, that poorly differentiated OCSCC was independently associated with unfavorable 5‐year outcomes––the only exception being LC. Collectively, these data indicate that, in addition to ENE, poor tumor differentiation is the second most relevant adverse risk factor for patients with pT3–4 OCSCC. In light of our findings, we suggest that the NCCN guidelines should include poor tumor differentiation as an adverse RF to further refine and tailor clinical management.

The current version (2017) of the World Health Organization (WHO) Classification of Head and Neck Tumors supports a simple grading system for OCSCC based on the Broders standard (i.e., well, moderately, and poorly differentiated tumors).^32^, ^33^ In 1980, Rapidis et al.^34^ (*n* = 136) have shown that patients with poorly differentiated OCSCC have less favorable prognosis than those with well differentiated tumors; additionally, they reported a direct correlation between the degree of tumor differentiation and patient survival. However, other studies failed to demonstrate a significant association between the WHO grading system and survival outcomes for patients with OCSCC (Table 5).^9^, ^10^, ^11^, ^12^, ^13^, ^14^, ^17^, ^20^, ^22^ Conversely, these investigations identified numerous other pathological parameters as significantly associated with prognosis––including budding,^11^, ^12^, ^13^, ^14^ budding and poor differentiation,^9^ budding and small nest size,^17^ budding and DOI,^20^ cohesion and smooth muscle actin,^10^ as well as worst pattern of invasion (WPOI) and perineural invasion.^22^ It is noteworthy that these studies were chiefly based on OS as the outcome of interest,^10^, ^11^, ^12^, ^13^, ^14^, ^17^, ^20^ whereas cancer‐specific survival and DFS were not specifically taken into account. In more recent investigations focusing on DFS and DSS, poor tumor differentiation was embedded in a more complex variable (termed budding grade III)^9^ and was identified as an independent prognostic factor.^22^ In this scenario, further clarification of the prognostic significance of this variable can assist in the optimization of risk stratification for patients with OCSCC.

**TABLE 5:** Summary of published studies focusing on the prognostic significance of traditional and non‐traditional pathological risk factors in patients with oral cavity cancer

Authors (years of recruitment)	Subsite‐specific stage (number)	Pathological variables (%)	Independent adverse risk factors (multivariable analysis)		
			Disease‐free survival	Disease‐specific survival	Overall survival
Elseragy et al.^9^ (1979–2010)	Tongue cT1–2N0 (311)	Poor differentiation: 24%, perineural invasion: 14%,^a^ budding grade: III, 47%	Budding grade III, >60 years old	Budding grade III, >60 years old	—
Marsh et al.^10^ (1992–2005)	Oral cavity (282)	Poor differentiation: nr, ENE: nr, SMA: high, nr, cohesion: nr, positive ≤1 mm: nr	**—**	—	High SMA, cohesion, age
Almangush et al.^11^ (1979–2009)	Tongue T1–2N0 (233)	Poor differentiation: 18%, budding ≥5: 35%, WPOI: nr	**—**	—	Budding ≥5, DOI ≥4 mm, High‐risk WPOI
Wang et al.^12^ (1996–2005)	Tongue (230)	Poor differentiation: nr, budding ≥5: 48%	**—**	—	Budding ≥5, pT3–4
Jensen et al.^13^ (nr)	Tongue, mouth floor (199)	Poor differentiation: 11%, margin+: 34%, ENE: 8%, budding > median: 50%	**—**	—	Budding > median, pStage III–IV, old age
Xie et al.^14^ (2001–2010)	Tongue pT1–2N0 (195)	Poor differentiation: 4%, budding: high intensity, 53%	—	—	High intensity budding
Boxberg et al.^17^ (2007–2012)	Oral cavity (157)	Poor differentiation: 25%, perineural: 26%, lymphovascular: 22%,^b^ new grade 3: 16%	—	—	pN+, new grade 3, age
Sawazaki‐Calone et al.^20^ (1998–2008)	Oral cavity (113)	Poor differentiation: 12%,^c^ BD score: high, 43%, margin <5 mm: 4%	—	—	High BD score, pT3–4
Lindenblatt et al.^22^ (1999)	Oral cavity (53)	Poor differentiation: 13%,^d^ high histological risk: 43%	Poor differentiation, high histological risk	High histological risk	High histological risk

Abbreviations: DOI, depth of invasion; ENE, extranodal extension; nr, not reported; SMA, smooth muscle actin; WPOI, worse pattern of invasion.

^a^ Budding grade: defined as poor differentiation either with or without ≥5 buds.

^b^ New grade: budding and small nest size.

^c^ BD score: tumor budding and depth of invasion.

^d^ Histological risk: lymphocyte infiltrate, worst pattern of invasion, and perineural invasion.

The ‘Protocol for the Examination of Specimens from Patients with Cancers of the Lip and Oral Cavity’ released from the College of American Pathologists suggests the adoption of a three‐level grading system (well, moderately, and poorly differentiated tumors)––in which selecting either the most prevalent grade or the highest grade is acceptable.^31^ Notably, when the most prevalent grade is selected, the proportion of poorly differentiated tumors is generally low. Conversely, upon selection of the highest grade, the proportion of poorly differentiated tumors tends to increase.

In the published literature, the prevalence of poorly differentiated OCSCC has been reported to range from 4% to 36% (Tables 5 and 6). Of the 11 studies focusing on the prognostic significance of traditional pathological RFs (Table 6), only three (including the present investigation) have separately analyzed clinical outcomes at the local, regional, and distant sites.^6^, ^7^ On examining patients with pT1–2N0 OCSCC, we have previously shown that poor differentiation is an independent adverse RF for NC.^7^ This study is the first to analyze the prognostic impact of poor differentiation in patients with pT3–4 OCSCC. Notably, we found that this variable was an independent RF for NC, DM, and all survival endpoints. In an analysis conducted in 18,115 patients as part of the Surveillance, Epidemiology, and End Results (SEER) study, poorly differentiated OCSCC was identified as an independent prognostic factor for DSS; however, separate data for local, regional, and distant sites were not available.

**TABLE 6:** Summary of published studies focusing on the prognostic significance of traditional clinicopathological risk factors in patients with oral cavity cancer

Author (years of recruitment)	Subsite‐specific stage (number)	Pathological variables (%)	Independent adverse risk factors (multivariable analysis)					
			Local control	Neck control	Distant metastases	Disease‐free survival	Disease‐specific survival	Overall survival
Liao et al. (1996–2018) current study	Oral cavity pT3–4 (1179)	Poor diff.: 11%, perineural invasion: 43%, lymphatic invasion: 8%, vascular invasion: 4%, margin+: 2%, ENE: 32%	ENE, positive margins	Poor diff., ENE	Poor diff., ENE Perineural invasion	Poor diff., ENE, positive margins, lymphatic invasion	Poor diff., ENE, lymphatic invasion	Poor diff., ENE, lymphatic invasion, age ≥65 years old
Thomas et al.^4^ (2004–2008)	Oral cavity (18,115)	Poor diff.: 17%	—	—	—	—	Poor diff.	—
Lin et al.^5^ (2008–2018)	Oral cavity (2535)	Poor diff.: 5%, margins ≤1 mm: 3%, ENE: 14%	—	—	—	Poor diff., ENE	—	—
Chen et al.^6^ (2004–2009)	Oral cavity pStage III–IV (628)	Poor diff.: 7%, perineural invasion: 43%, lymphovascular invasion: 29%, positive/close margins: 37%, ENE: 31%	—	—	Poor diff., pN2C	Poor diff., pN+, positive/close margins	—	Poor diff., ENE, pN+, pStage IV, positive/close margins, male sex
Liao et al.^7^ (1996–2008)	Oral cavity pT1–2N0 (387)	Poor diff.: 5%, Perineural invasion: 12%, Lymphatic invasion: 0.5%, Vascular invasion: 0.5%	—	Poor diff., DOI ≥4 mm	—	Poor diff., DOI ≥4 mm	Poor diff., DOI ≥4 mm	Lymphatic invasion
Rodrigues et al.^8^ (1999–2006)	Tongue, mouth floor (380)	Poor diff.: 5%, Perineural invasion: 23%, vascular invasion: 13%, ENE: 7%	—	—	—	Perineural invasion, pStage III–IV	Poor diff., Perineural invasion, pStage III–IV	Perineural invasion, pStage III–IV
Noble et al.^15^ (1995–2013)	Oral cavity stage III–IV (191)	Poor diff.: 36%, perineural invasion: 36%, lymphovascular invasion: 32%, margin+: 20%, ENE: 39%	—	—	—	Poor diff., nodal ratio	—	—
Beenken et al.^16^ (1956–1994)	Tongue T1–2 (169)	Poor diff.: 7%, margin+: 1%	—	—	—	Poor diff.	—	—
Fang et al.^18^ (2003–2007)	Oral cavity (150)	Poor diff.: 8%, Perineural invasion: 27%, ENE: 19%	—	—	—	—	—	poor diff., ENE, pT3–4, age >51 years
Mahia et al.^19^ (1998–2003)	Oral cavity (118)	Poor diff.: 6%	—	—	—	Poor diff., pStage IV	—	—
Eskander et al.^21^ (1994–2008)	Upper gum, hard palate (97)	Poor diff.: 9%, Perineural invasion: nr, Lymphovascular invasion: nr, margin+: 13%	—	—	—	Poor diff., pT3–4	—	—

Abbreviations: diff., differentiation; DOI, depth of invasion; ENE, extranodal extension; nr, not reported.

In the current investigation, the 5‐year LC rates did not differ significantly according to tumor differentiation. Compared with patients with moderately and well differentiated tumors, those with poorly differentiated OCSCC had a higher frequency of certain pathological RFs––including positive margins, margins <5 mm, perineural invasion, lymphatic invasion, and vascular invasion. However, this was not found to have a significant impact in terms of LC. Conversely, the spread of poorly differentiated OCSCC to neck nodes increased the frequency of ENE (which may be as high as 61%)––which in turn portends an increased risk of distant metastases and less favorable NC. While the adverse prognostic impact of ENE is widely recognized, we also found that the concomitant presence of poor differentiation and ENE was associated with less favorable 5‐year NC, DM, DFS, and DFS rates compared with ENE alone. This suggests that a thorough evaluation of tumor differentiation may further improve both prognostic stratification and treatment selection in patients with OCSCC.

Margin status and lymph node metastases are the main prognostic determinants in patients with oral cavity cancer. As for margin status, positive margins have been widely associated with less favorable clinical outcomes compared with close margins. While the NCCN guidelines have consistently considered ENE and positive margins as major adverse prognostic factors, a close margin (<5 mm) has been recognized as a poor prognosticator as of 2020 only. In this study, we identified a margin status as an adverse risk factor for LC (*p *= 0.001) and DM (*p* = 0.011)––with significant adverse implications for DFS (*p *= 0.001), DSS (*p* = 0.002), and OS (*p *= 0.005; Table 2). However, after adjustment for potential confounders in MVA, the positive margin retained its independent prognostic significance for LC and DFS only (Table 3). These results demonstrate that patients with pT3–4 OCSCC and positive margins tended to relapse at local and distal––rather than regional––sites. This result is consistent with other findings independently reported by other head and neck research groups (Memorial Sloan Kettering Cancer Center, Tata Memorial Center, and MD Anderson Cancer Center) that failed to identify the margin status as an independent adverse prognostic factor for NC.^1^

This cohort study has several limitations. First, the single‐center design of our study may have limited the external validity of the results; in this scenario, independent replication of our findings is necessary before advocating the inclusion of poor tumor differentiation as an adverse risk factor in the NCCN guidelines. Second, our research was undertaken in a betel quid chewing endemic area and––for that reason––our conclusions might not be generalizable to Western countries. Notably, in this study, betel quid chewers tended to have a lower frequency of poorly differentiated OCSCC (10.3% [102/986]) than non‐chewers (13.5% [26/193], *p* = 0.206). Another limitation pertains to the homogeneous treatment––which was based on surgery either with or without adjuvant therapy. This caveat may hamper the extension of our findings to patients who did not initially undergo primary tumor excision.

Despite these limitations, our data represent a promising step in understanding the prognostic role of poor tumor differentiation in patients with locally advanced OCSCC. Patients with poorly differentiated tumors tended to relapse at regional and distal sites and showed less favorable survival endpoints. Notably, poor tumor differentiation was identified as the most unfavorable prognostic variable following ENE. While our findings may have significant implications for the clinical management of patients with poorly differentiated OCSCC, further research is needed to replicate these results in other geographic areas, as well as to clarify mechanisms, to examine more rigorously the hypothesis of a synergy between poor tumor differentiation and ENE, and to identify tailored treatment approaches.

## CONFLICT OF INTEREST

The authors declare no conflict of interest.
